# Beneficial Effects of *Opuntia humifusa* (Korean Cheonnyuncho) on Human Health Based on Antioxidant Properties: Systematic Review and Meta-Analysis

**DOI:** 10.3390/antiox12010174

**Published:** 2023-01-11

**Authors:** Le Thi Nhu Ngoc, Ju-Young Moon, Young-Chul Lee

**Affiliations:** 1 Department of Industrial and Environmental Engineering, Graduate School of Environment, Gachon University, 1342 Seongnam-Daero, Sujeong-gu, Seongnam-si 13120, Gyeonggi-do, Republic of Korea; 2 Department of Beauty Design Management, Han-Sung University, 116 Samseongyoro-16gil, Seoul 02876, Gyeonggi-do, Republic of Korea; 3 Department of BioNano Technology, Gachon University, 1342 Seongnam-Daero, Sujeong-gu, Seongnam-si 13120, Gyeonggi-do, Republic of Korea

**Keywords:** *Opuntia humifusa* (OH), antioxidant activities, skin aging, cancer, diabetes

## Abstract

In this study, a systematic review and meta-analysis are conducted to evaluate the medical applications of *Opuntia humifusa* (OH). A total of 171 articles are peer-reviewed; however, only 15 individual studies were included after the manual screening to eliminate unnecessary studies. A comparative standardized means difference (SMD) between the OH and control groups is used as a parameter to demonstrate the beneficial effects of OH for skin aging, cancer, and diabetes treatments based on its antioxidant activities. The OH treatment exhibits positive results in improving collagen synthesis and demonstrates that it is nontoxic to normal human cells without analytical heterogeneity (SMD = 1.18 [0.11, 2.26] and I^2^ = 0%). Moreover, the results confirm the effectiveness of OH treatment on downregulatory cancers in terms of decreased cancer cell proliferation and tumor weight and increased numbers of cancer cells in the apoptosis phase (pooled SMD = −1.17 [−1.72, −0.62]). However, this comparison does not yield a statistically significant result (I^2^ = 69%). Additionally, the OH treatment is found to reduce the symptoms of diabetes in diabetic rats, particularly by lowering glucose and triglyceride levels and increasing high-density lipoprotein cholesterol levels. This study suggests that OH extracts are helpful for the prevention of human diseases and might be potential candidates for future medicines.

## 1. Introduction

There is no denying that traditional medicine using natural plant extracts has gained worldwide acceptance with many favorable factors, such as diversity, easy access, relatively low cost, and shallow side effects compared with synthetic drugs. Plants and their phytochemicals, especially *Opuntia* species (spp.), are essential in improving human health care. *Opuntia* spp. are the most abundant of the Cactaceae family and have shown many ideal properties and a high biotechnological capacity in medicine [1,2]. Notably, *Opuntia* spp. (approximately 126 species) are distributed in America, Africa, Asia, Australia, and the central Mediterranean area. The wildest species are *O. streptacantha*, *O. hyptiacantha*, *O. albicarpa*, *O. megacantha*, *O. ficus-indica*, and *O. humifusa* (OH), with potential medical and economic effects [2]. For instance, *O. ficus-indica* is as vital as corn and tequila agave in the agricultural economy of Mexico and represents important food and feed resources. *O. streptacantha* and *O. robusta* show hepatoprotective effects against acetaminophen-induced acute liver damage [3]. The novel pectin obtained from the peel of *O. albicarpa* shows non-Newtonian rheological behavior and gelling ability in the presence of calcium ions, suggesting new natural polysaccharides for diverse applications such as personal care products, food, and nanomaterials in drug delivery [4]. *O. humifusa* (OH) has been widely applied for topical cosmetic, biomedical, and food-additive purposes [5,6,7,8].

OH, known as Korean Cheonnyuncho, is cultured primarily in Korea, even in cold environments below −20 °C [2,9]. OH is a low prostrate succulent, forming clumps usually only one or two stem segments tall [10]. The green stem segments are fleshy and sparsely covered with barbed bristles. The flowers that appear in June are large and yellow and are found along the edges of mature segments [10]. The juicy red or pink fruits measure from 3–5 cm. At maturity, the fruit color changes from green to red or pink and usually remains on the cactus until the following spring [10]. The rich nutrients in OH (such as betalains, minerals, vitamins, flavonoid compounds, phenolic compounds, and polysaccharides) have been well-documented [11]. Notably, betalains are water-soluble nitrogen-containing pigments and are identified in OH fruits as betanidin, betanin, isobetanidin, isobetanin, neobetanin, phyllodcactin, and gomphrenin [2]. These betalains are potential radical scavengers with antioxidant activity 3–4 times higher than ascorbic acid, catechin, and rutin [2]. OH stems have higher concentrations of total flavonoids and phenolics (4–90 mg/mL) compared with other cactus species [11]. Yoon et al. (2009) reported that the vitamin C content in an OH stem is approximately 260.94 mg/100 g, which is higher than that in *Opuntia ficus-indica* [12]. Kwon et al. (2012) and Kang et al. (2013) found abundant minerals, such as calcium (Ca), magnesium (Mg), iron (Fe), sodium (Na), and potassium (K), in OH stems and fruits [13,14]. The abundance of natural antioxidants in these plants, primarily in their phenolic and flavonoid compounds, is believed to contribute significantly to their antioxidant capacity.

Many studies have investigated the beneficial effects of OH based on its ideal antioxidant properties [5,6,7,8]. For example, Moon et al. (2020) suggested that microwave-assisted OH extract can act as a potential cosmeceutical ingredient owing to its ability to prevent particulate matter-induced skin oxidative stress and inflammation [5]. Halm et al. (2010) demonstrated that the water extractions of OH suppress the proliferation of U87MG glioblastoma cancer cells [15]. Halm et al. (2011) reported that oral administration of OH in diabetic rats significantly decreases both total cholesterol and low-density lipoprotein cholesterol (LDL-C) but increases high-density lipoprotein cholesterol (HDL-C), thus suggesting potential hypolipidemic activity [11].

Although several studies have indicated the contributions of OH to the limitation and prevention of human diseases (such as skin aging, cancer, and diabetes), studies providing overall assessments and systematic reviews of the beneficial effects of OH on human health are lacking. Thus, this study reviewed individual studies and performed a systematic review and meta-analysis to assess the statistically significant therapeutic benefits of OH. Herein, the standardized mean differences (SMDs) between OH-treated and control groups were collected and analyzed from three perspectives (skin aging, cancer, and diabetes). The comparative results may contribute to identifying the beneficial effects of OH on human health based on its antioxidant properties.

## 2. Materials and Methods

### 2.1. Literature Search Strategy

According to the “Preferred Reporting Items for Systematic Reviews and Meta-Analysis” (PRISMA) protocol 2009 [16], a literature search on the beneficial effects of OH on human health based on its antioxidant properties was performed to obtain relevant articles published up to 2022. The registration number is CRD42023387950, which has been approved by PROSPERO on 15 January 2023. The English-language databases included the Cochrane Library, PubMed, Elsevier, EMBASE, and MedRxiv. The search was performed using the keywords: OH, Korean Cheonnyuncho, antioxidant, anti-diabetic, anti-cancer, anti-aging, photo-aging, cosmetic benefits, and human health.

### 2.2. Study Selection and Data Extraction

#### 2.2.1. Study Selection

All studies investigating the benefits of using OH to improve human health, particularly its anti-diabetic, anti-cancer, and skin health benefits, were included in this study. All in vitro and in vivo studies involving at least one treatment subject and one matched issue in the control group were considered, as long as the positive effects of OH on human health did not exhibit any differences. Individual studies with multiple arms of the investigation were deemed eligible if they directly compared the treatment and the control groups with the right components included in the meta-analysis. In the in vitro and in vivo studies, the results were reported as the mean ± standard difference (SD), regardless of whether it was the primary outcome. No restrictions were set on the treatment-control groups, experimental designs, types of OH used, extraction methods, or geographical regions. All included articles were written in English or Korean; articles in Korean were translated into English for easy data extraction.

Studies on applications of OH in other fields, such as the food industry, were excluded. Articles that were entirely or partially duplicated from different studies were also excluded. Among repeated articles, only the latest article was included. Conference abstracts, presentations, reviews, and primary science manuscripts were excluded from this systematic review.

#### 2.2.2. Data Extraction

Three independent reviewers screened each included study and extracted data for the systematic review and meta-analysis. The data extracted from each in vitro and in vivo study were the first author’s name, year of publication, region of study, parts of OH (fruit, stem, seed, and root), extraction method, types of diseases and conditions considered (anti-cancer, anti-diabetic, anti-aging), study design (human cells and animal), and primary outcomes (mean ± SD values).

### 2.3. Meta-Analysis

For all continuous outcomes, the mean and SD of each study’s preliminary results in both the OH treatment and control groups were pooled using the radon effects model and expressed as the SMD with a 95% confidence interval (CI) [17]. The SMD was obtained by dividing the mean difference between the two groups by the pooled variance and adjusting for small samples. If the value 0 was not within the 95% CI, then the pooled SMD was statistically significant at the 5% level. SMDs of approximately 0.2 or less were considered as small, 0.5 as moderate, and 0.8 or greater as large [17]. The extent to which the observed variability between studies was owing to actual differences was quantified using the I^2^ statistic. Heterogeneity was considered small when I^2^ was less than 25%, moderate when it was 25–50%, and large when it was greater than 50% [17]. A value of *p* ≥ 0.1 was considered significant, and bias was examined using a funnel plot. A subgroup analysis assessed the overall effects in the subgroups according to the raw material of treatment, concentration of treatment, extraction methods, and type of study design.

All analyses were performed using Review Manager [18] (version 5.3, Copenhagen: The Nordic Cochrane Centre, The Cochrane Collaboration, 2014).

## 3. Results

### 3.1. Characteristics of Included Studies

**Figure 1:**
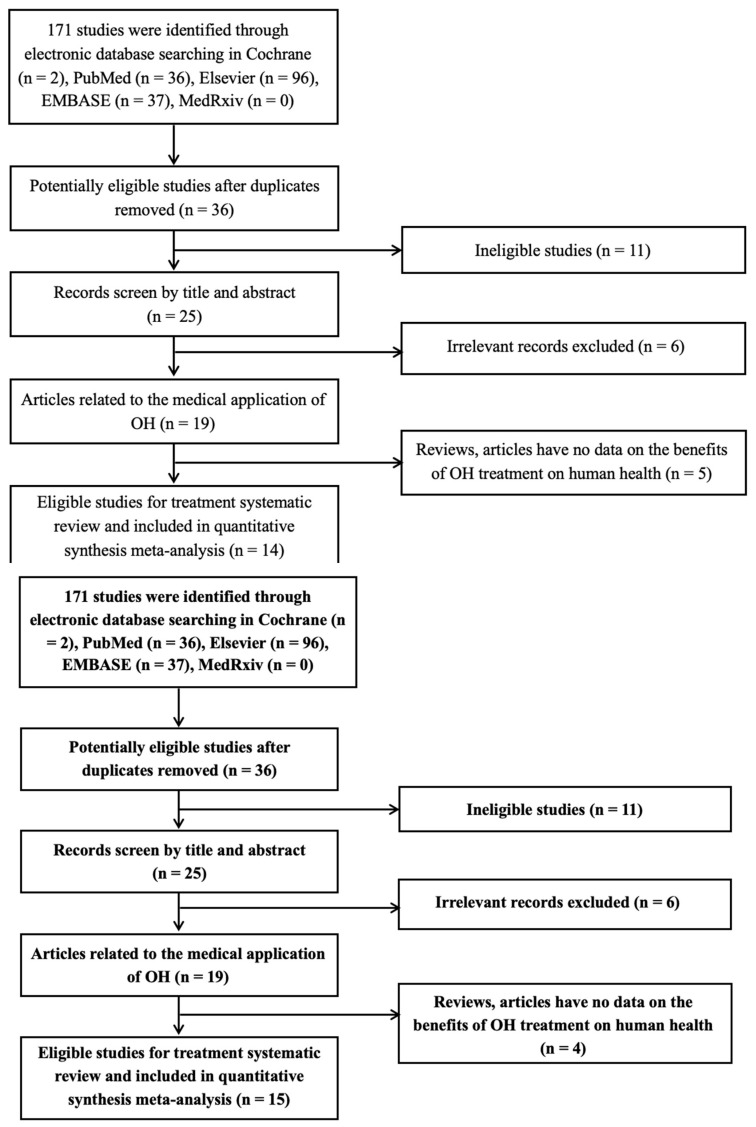
Systematic screening stages of literature review on *Opuntia humifusa* (OH).

Figure 1 shows a flowchart of the process by which the qualified articles were screened. In this study, 171 publications were screened by searching their titles and abstracts from the aforementioned databases. After carefully reviewing these articles on the beneficial effects of OH on human health (inhibiting cancer and diabetes and controlling skin aging) based on its antioxidant properties, individual studies were selected for the quantitative meta-analyses.

#### Description of Included Studies

The main characteristics of the 15 studies are summarized in Table 1. Among the fifteen articles, four studies reported a positive association between the optical application of OH and reductions in skin aging [5,19,20,21], nine studies found anti-cancer benefits [6,7,15,21,22,23,24,25,26], and three studies demonstrated the beneficial effects of OH in reducing symptoms of diabetes [9,11,13,14,27]. OH species are cultivated primarily in Korea; thus, all of these studies were performed only in Korea, covering the period from 2005 to 2020. The OH materials used in these studies were processed from different parts of the OH, including the fruit (n = 6) [3,4,6,9,10,13], stem (n = 9) [5,7,9,11,13,21,22,24,25], and peel (n = 1) [21]. Subsequently, the raw OHs were extracted into a solution using various solvents (including methanol, ethanol, hexane, acetone, ethyl acetate, and water) for in vitro studies on human and animal cell lines. Moreover, the raw OHs were blended and freeze-dried into a powder for oral administration [11,13]. Notably, one study performed extraction based on a microwave-assisted extraction method that produced greater amounts of antioxidant compounds and yielded a greater extraction efficiency [5]. Most studies reported high antioxidant compounds (polyphenolic and flavonoids) and other physicochemical compositions (such as moisture, ash, carbohydrate, crude protein, crude fate, and minerals). The studies had three major experimental subject types: normal human cells (human keratinocyte (HaCaT) and human dermal fibroblast (HDF) cells), human cancer cells (RAW 264.7, U87MG, HeLa, AGS, MCF-7, and MC3T3-E1), and mice. These subjects were treated with various concentrations of OH extracts (100–500 μg/mL) and were further studied using in vitro and in vivo experiments. To increase the statistical significance of the meta-analysis, data was extracted under various types of outcomes for each analysis aspect, such as “cell viability (%), relative protein expression, and collagen synthesis (%),” “cell viability (%), cell death on apoptosis (%), and tumor weight (g),” and “body weight (g), cholesterol (mg/dL), triglyceride (mg/dL), glucose (mg/dL), insulin (ng/mL)” for the anti-aging, anti-cancer, and anti-diabetic effects, respectively.

### 3.2. Quality Assessment of Included Studies

**Figure 2:**
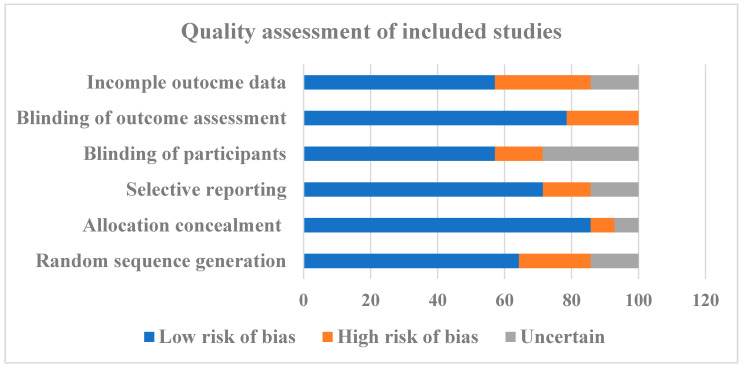
Risk of bias in individual studies graph.

The quality of the eligible studies was assessed by considering the bias in the random sequence generation, selective reporting, allocation concealment, blinding of participants, blinding of outcome assessment, and incomplete outcome data, following the Cochrane guidelines [18]. These criteria were evaluated at three levels (low risk, high risk, and uncertain) to indicate a lack of information and uncertainty over the potential for bias. The results indicated that almost all the criteria exhibited a low risk of bias, thus resulting in an evident enhancement of the statistical significance of the meta-analysis (Table 2 and Figure 2).

### 3.3. Beneficial Effects of Opuntia Humifusa (OH) on Improving Human Health

#### 3.3.1. Controlling Skin Aging

**Figure 3:**
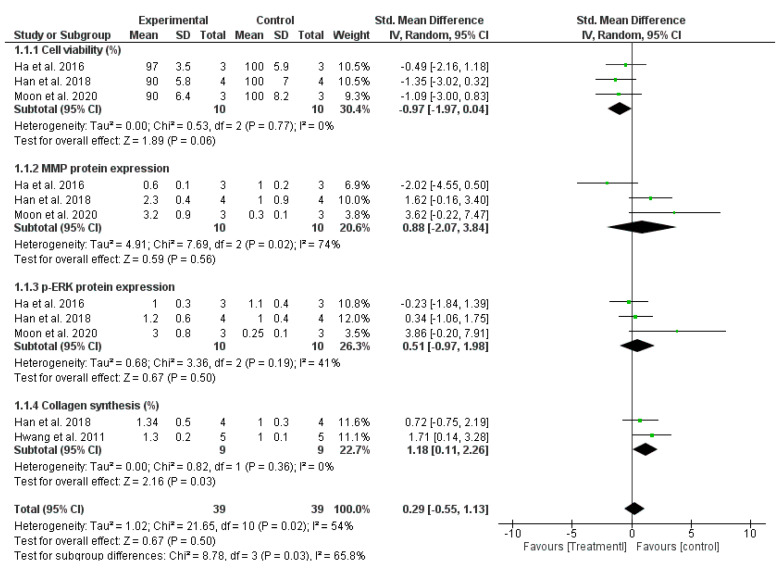
Comparison of reducing skin-aging signal between OH treatment and control groups [5,19,20,21].

The positive effect of OH on skin aging was assessed based on a comparison of the mean ± SD in four individual studies with three sub-criteria: cell viability (%) of normal human skin cells (HaCaT and HDF cells), protein expression levels (matrix metalloproteinase (MMP) and phospho-extracellular signal-regulated kinase (p-ERK) proteins), and collagen synthesis (%). The SMDs of the subgroup analyses are presented in Figure 3 and Table 3. A random effects model was used to calculate the pooled SMD because substantial heterogeneity was found between the studies (I^2^ = 54% and *p* = 0.02). The pooled SMD exhibited no significant difference between the treatment and control groups (SMD = 0.29 [−0.55, 1.13]), regarding the anti-aging properties of OH. Although each study reported an increase in the expression of MMP and p-ERK proteins, the meta-analysis indicated that the OH treatment did not entirely upregulate the expression of proteins related to the anti-aging mitogen-activated protein kinase (MAPK) pathway, with SMDs of 0.88 [−2.07, 3.84] (I^2^ = 74%) and 0.51 [−0.97, 1.98] (I^2^ = 41%), respectively. The cell viability of normal human cells did not differ significantly between the two groups, indicating that the OH treatment was nontoxic and showed high biocompatibility (SMD = −0.97 [−1.97, 0.04] and I^2^ = 0%). However, a positive association was observed between OH treatment and enhancement of collagen synthesis, which plays an essential role in the anti-aging pathway (SMD = 1.18 [0.11, 2.26]).

#### 3.3.2. Inhibition of the Growth of Cancers

**Figure 4:**
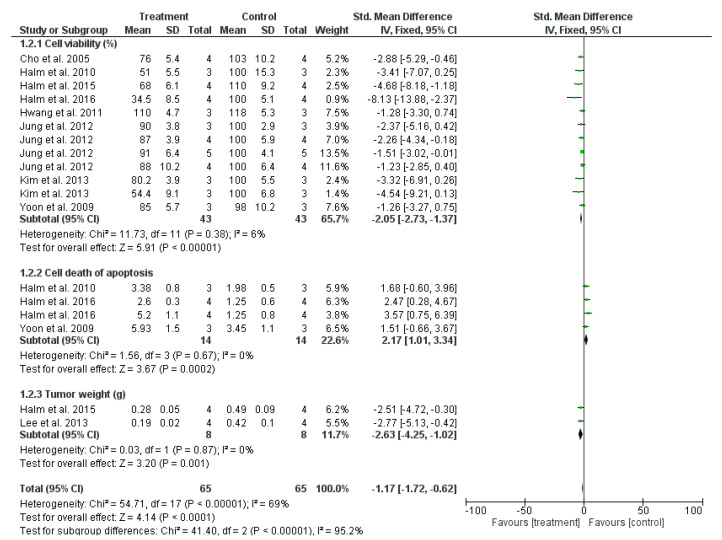
Comparisons of inhibiting the growth of cancer between OH treatment and control groups [6,7,15,21,22,23,24,25,26].

The pooled SMD of nine studies on the downregulation of cancer growth using OH extraction was assessed based on a subgroup analysis of three sub-criteria: cancer cell viability (%), cell death owing to apoptosis, and tumor weight (g) (Figure 4). Although the pooled SMD from the three comparisons exhibited a significant difference between the treatment and control groups (SMD = −1.17 [−1.72; −0.62]), considerable heterogeneity was observed in the entire subgroup analysis (I^2^ = 69% and *p* < 0.00001). Each comparison was based on a distinguished data unit; thus, heterogeneity was inevitable. However, the heterogeneity did not exist when considering each comparison, with I^2^ = 6% and 0%. The results indicated that OH could significantly kill the cancer cells; particularly, it reduced the viability of cancer cells compared with the control group (SMD = −2.05 [−2.73, −1.37], I^2^ = 6%, and *p* = 0.38). The meta-analysis also yielded a considerable SMD value in the analysis of the cell death in the apoptosis phase (%) between the treatment and control arms (Table 3), without heterogeneity. The increase in cell death during the apoptosis phase demonstrated that OH treatment promoted cancer cell death and inhibited cancer development. In addition, the OH treatment strongly decreased the weights of tumors, compared with the control tumor samples, with an SMD of −2.63 [−4.25, −1.02] (I^2^ = 0% and *p* = 0.87).

#### 3.3.3. Downregulation of Diabetes

**Figure 5:**
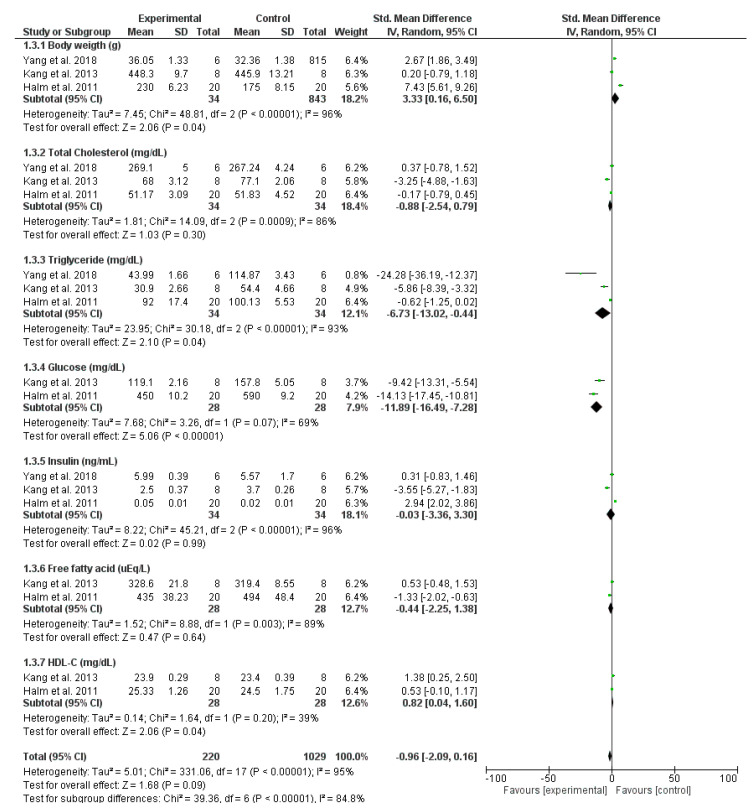
Comparisons of inhibiting the development of diabetes between OH treatment and control groups [9,11,13].

Several comparisons were performed to compare the mean differences between the treatment and control groups for various laboratory parameters of diabetic disease, including body weight (g), total cholesterol (mg/dL), triglycerides (mg/dL), glucose serum (mg/dL), insulin serum (ng/mL), free fatty acids (FFA) (μEq/L), and high-density lipoprotein values (HDL-C) (mg/dL) (Figure 5 and Table 3).

The body weights of the mice significantly increased after treatment with OH relative to those of untreated mice (SMD = 3.33 [0.16, 6.50]); however, the analysis did not provide high statistical significance owing to its heterogeneity (I^2^ = 96% and *p* < 0.00001). Regarding the cholesterol-lowering effects of OH, differences in the triglyceride and cholesterol levels of the participating mice were compared. Evidently, oral administration of OH decreased triglyceride lipid and increased HDL-C levels in the administrated group, compared with the control group (−6.73 [−13.02, −0.44] and 0.82 [0.04, 1.60], respectively). However, the cholesterol lipid levels were not significantly different between the treated and untreated groups (SMD = −0.88 [−2.54, 0.79]). The blood glucose-lowering effects of OH were demonstrated through the ability of OH to reduce glucose levels in treated mice relative to the control mice; however, the results did not achieve high statistical significance (−11.89 [−16.49, −7.28], I^2^ = 69%, and *p* = 0.07). In addition, OH treatment did not considerably alter the regulation of serum insulin and FFA levels in diabetic mice. Overall, the oral administration of OH can prevent the weight loss caused by diabetes and effectively lower the glucose and triglyceride levels in diabetic patients.

### 3.4. Subgroup Analysis

**Figure 6:**
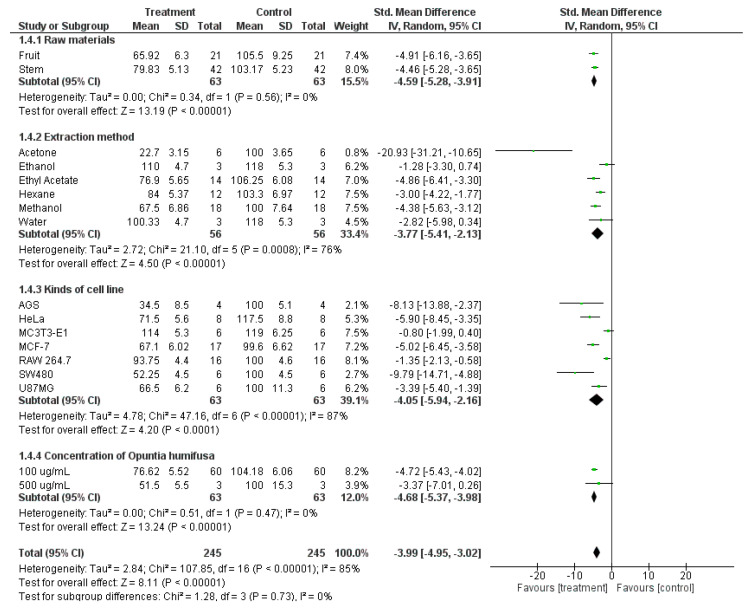
Subgroup analysis for the reduction of cancer cell proliferation based on four sub-criteria: raw materials, extraction methods, types of cancer cell lines, and concentrations of OH treatment.

The anticancer properties of OH were reported in various studies with significant differences in experimental conditions (e.g., cell lines, raw materials, and extraction methods); thus, the meta-analysis might have led to underestimated results. Therefore, a subgroup analysis was designed to assess the ability of OH to prevent cancers based on the cell viability values (%) of cancer cells treated with OH (Figure 6 and Table S1). First, the subgroup analysis was stratified based on the raw materials pooled as fruit or stem. Second, the included studies were classified based on the OH extraction methods, including methanol, ethanol, ethyl acetate, hexane, acetone, and water. Third, the type of cancer cell line was considered as a category for the subgroup analysis. The included studies were performed on several cell types, including cervical cancer cells (HeLa), gastric cancer cells (AGS), osteoblastic cells (MC3T3-E1), breast cancer cells (MCF-7), macrophage-like cells from mouse tumors (RAW 264.7), human colon adenocarcinoma cells (SW480), and human glioblastoma cells (U87MG). Finally, these studies were classified based on the concentrations of the OH extractions (100 μg/mL and 500 μg/mL).

The subgroup analysis was performed using random effect models because some comparisons had considerable heterogeneity (I^2^ > 50%). According to the pooled SMDs, the OH treatment exhibited a significant difference in cell viability between the treatment and control groups for all sub-criteria. The comparisons regarding raw materials and concentrations of OH extraction yielded a high statistical significance without heterogeneity, with SMDs of −4.59 [−5.28, −3.91] (I^2^ = 0% and *p* = 0.56 > 0.1) and −4.68 [−5.37, −3.98] (I^2^ = 0% and *p* = 0.47 > 0.1), respectively. By contrast, heterogeneity was observed in the comparisons based on the extraction methods and types of cell line categories (I^2^ = 76% and 87%, respectively).

## 4. Analysis Bias

**Figure 7:**
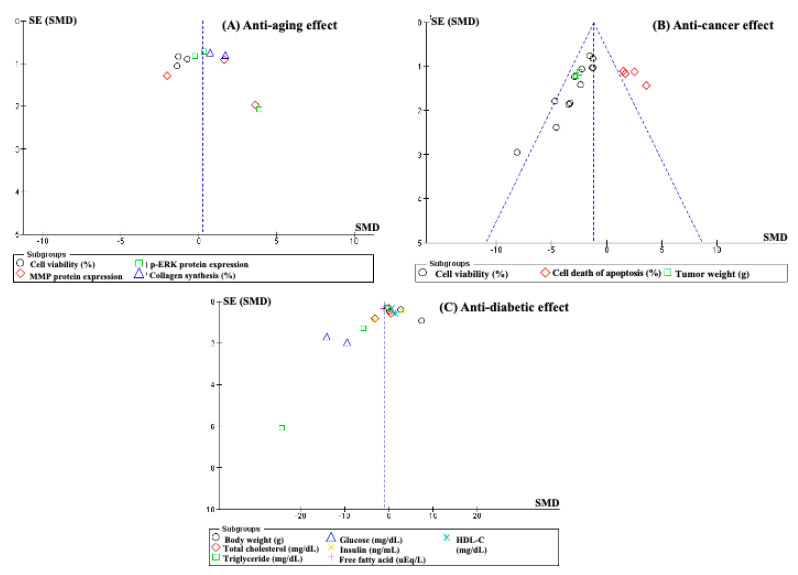
Publication bias of included studies for the effectiveness of using OH for the treatment of skin aging, cancer, and diabetes.

Funnel plots were constructed to detect publication biases. The results indicated that each analytic criterion’s outcomes were published similarly with a positive or negative association between the treatment and the control groups, thus leading to asymmetrical publication biases (Figure 7). In addition, the number of comparisons was small, which might have caused underrated meta-analysis results and publication biases. Therefore, this meta-analysis should be repeated with numerous prospective studies to reduce heterogeneity and publication bias.

## 5. Discussion

In this study, the beneficial effects of OH treatment on human health were assessed through a systematic review and meta-analysis of 15 in vitro and in vivo studies. In terms of physicochemical components, the OH extractions contained high phenolic and flavonoid contents (4–90 mg/mL) as well as various minerals (Cu^2+^, Fe^2+^, Ca^2+^, Mg^2+^, Na^+^, and K^+^), despite the use of different extraction methods. This suggests that OH treatment with high antioxidant activities could be a potential agent for cosmetic and medical applications. The potential benefits of OH in the medical field were assessed using meta-analyses. The results revealed that OH treatment is ideal for controlling skin aging and regulating cancer and diabetes.

Aging results from progressive physiological changes that reduce biological functions and an organism’s capacity to adapt to metabolic stress over time. Aging occurs via two general pathways: intrinsic and extrinsic [28]. Intrinsic skin aging is attributed to the reduction of collagen I/III synthesis, which leads to a loss of elasticity and increased wrinkling, thus making the skin more fragile [28,29]. External factors (smoking, pollutants, and UV irradiation) can generate a massive amount of reactive oxygen species (ROS), consequently inducing inflammation, decreasing collagen synthesis, and increasing collagen degradation. The intracellular mechanisms are attributed to the activation of MMPs, collagenases, and gelatinase, thereby causing skin damage [29]. Among the numerous anti-aging agents, antioxidants can effectively prevent symptoms related to photo-induced skin aging [5,30]. In particular, flavonoids and phenolic compounds can vigorously protect DNA against the damage caused by ROS radicals and avoid ROS radicals generation via complexation with metal ions (Cu^2+^ and Fe^2+/3+^) [5]. Betalains also play a vital role in antioxidant activity by scavenging ROS, galvinoxyl, hydroxyl, and superoxide free radicals, subsequently preventing DNA damage [2,31]. Notably, the antioxidant capacity of betaxanthins is related to the presence of one or two phenolic hydroxyl groups in their structure [2]. Betacyanin can inhibit the production of nitrogen radical or nitrogen oxide (NO) species regarding the presence of a catechol group in the betanidine structure [2]. Subsequently, they can promote collagen production, stimulate the production of new skin cells, and inhibit melanin production, thereby improving anti-aging signs and skin appearance. In this study, the effectiveness of OH for controlling skin aging was assessed from three perspectives: cell viability, MMP and p-ERK protein expressions, and collagen synthesis. These results indicated that OH treatment induced a significant difference in collagen synthesis (%) between the two groups (Table 3). In addition, the OH treatment did not significantly reduce the viability of skin cells relative to that of the control group; that is, OH was not toxic to skin cells at a low concentration (approximately 100 μg/mL) (Table 3). Thus, the antioxidants in the OH extractions play significant roles in promoting collagen synthesis, reducing inflammation, and inhibiting DNA damage, thus making them effective in anti-aging therapy.

Cancer development is a multistage process that involves a series of individual steps, including initiation, promotion, progression, invasion, and metastasis. Notably, it is characterized by an accumulation of alterations in cancer-regulating genes, such as oncogenes and tumor suppressor genes, thus resulting in altered cellular processes, such as decreased apoptosis, increased proliferation, and cell maturation and differentiation [32]. Anticancer agents, including antioxidants, exert inhibitory effects on carcinogenesis and tumor growth via two primary mechanisms: modifying the redox status and interfering with essential cellular functions (cell cycle, apoptosis, inflammation, angiogenesis, invasion, and metastasis) [7,32]. First, these antioxidants can scavenge and reduce ROS production and act as transition metal chelators; thus, they can inhibit carcinogen/toxin-induced cellular oxidative damage and exert significant chemopreventive activities [32]. In addition, phenolics and flavonoids may hinder the formation and growth of tumors by inducing cell cycle arrest during different cell phases (G1, S, S-G2, and G2), such as upregulating the expression of p21, p27, and p53 and downregulating cyclin D1 and cyclin-dependent kinases (CDK4) [33]. Betalains impede inflammatory lesion development by limiting inflammatory cell invasion, releasing hypochlorous acid (HClO), and neutralizing ROS production induced by tumor cells and inflammatory cells, thereby reducing oxidative stress [31]. Therefore, the decreased proliferation observed in cancer cells and tumor growth inhibition correlates with the growth arrest typically occurring when cultured cancer cells are treated with antioxidant compounds [7]. Apoptosis-inducing agents are expected to be ideal anticancer drugs because they eliminate severe cell damage and tumor cells. The induction of apoptosis includes induction of cell cycle arrest; blocking of ERK, c-Jun N-terminal kinase (JNK), and p38 MAPK pathways; inhibition of the activation of transcription factors, NF-kB, and activator protein-1 (AP1); suppression of protein kinase C (PKC); and suppression of growth factor-mediated pathways [32]. The meta-analysis in this study confirmed the ability of OH to reduce the cell viability of cancer cells, inhibit tumor growth, and increase the apoptosis phases (Table 3). Thus, high amounts of betalains, phenolics, and flavonoids, as strong ROS scavengers, play critical roles in the anticancer properties of the OH extractions.

Diabetes is a group of metabolic disorders characterized by high blood sugar levels over a prolonged period. This disease is due to either the pancreas not producing enough insulin or the cells of the body not responding correctly to the insulin made [34]. Diabetes is characterized by increases in total cholesterol, LDL cholesterol, and triglyceride levels and a decrease in HDL-C. Diabetes induction is related to oxidative stress, glycosylation, and metabolic stress. [34]. Remarkably, the disease is accompanied by an increased formation of free radicals and decreased antioxidant capacity, leading to oxidative damage to cellular components [35]. Therefore, significantly reducing the total cholesterol, increasing the HDL-C levels, and scavenging ROS radicals play essential roles in inhibiting the development of diabetes. Antioxidant pharmacotherapy can effectively prevent oxidative stress and related diabetic vascular complications by inhibiting the intracellular free radical formation and increasing the antioxidant defense enzyme capabilities [35]. The intake of antioxidants can eliminate free ROS radicals, increase beta cell proliferation, and inhibit apoptosis in normal cells, thereby protecting against tissue damage in diabetes [36]. In this study, blood glucose and triglyceride lipid levels were found to be significantly ameliorated in the OH treatment group compared with the control group (Table 3). Moreover, the analysis confirmed the capacity of OH to increase the level of HDL-C in the blood, thus suggesting its potential effectiveness in anti-diabetic therapy. The hypothesis regarding the increased insulin levels via the administration of OH was not confirmed as no significant difference was observed between the administered and the control groups. The presence of heterogeneity and underestimated results could have resulted from the small size of the analysis data; therefore, the analysis should be repeated with a large sample size. Overall, the beneficial effect of OH treatment is in part due to the high amounts of antioxidants responsible for eliminating oxidative stress and preventing cell damage, thus resulting in the downregulation of diabetes.

## 6. Limitations of Study

The meta-analysis performed has three primary limitations. First, it included a small number of studies; this may have resulted in underestimated and highly heterogeneous outcomes. Second, because most in vitro studies were performed on various cell lines and under different experimental conditions, the meta-analysis evaluations may not be highly accurate, particularly from an inhibitory cancer perspective. Finally, the included studies were only conducted in Korea; therefore, statistical significance could not be determined from a geographical perspective, leading to low statistical significance on a global scale.

## 7. Conclusions

This study determined that OH is helpful for medical and cosmetic applications. A systematic review and meta-analysis revealed that the antioxidant compounds present in the OH extracts, as free radical scavengers, play essential roles in treating skin aging and inhibiting the growth of diabetes and cancer. The results revealed the beneficial effects of OH treatments from almost all evaluation perspectives: “improving collagen synthesis,” “reducing cancer cell proliferation and increasing apoptosis phase of cancer cells,” and “reducing glucose and triglyceride levels, increasing HDL-C levels, and preventing weight loss” for treatments of skin aging, cancer, and diabetes, respectively. However, the results did not yield high statistical significance owing to the current limitations. Therefore, future studies on the clinical application of OH are necessary to improve the statistical significance.

## Figures and Tables

**Table 1:** Summary of the characteristics of included studies.

References	Region	Raw OH	Extraction Methods	Physical Chemical Properties		Experimental Subjects	Main Outcomes
				Compounds	Mean ± SD		
Cho et al. 2005 [24]	Korea	Stem	Methanol, hexane, ethyl acetate, ethanol, and water extraction	—	—	In vitro experiments on RAW 264.7 cell line	Cell viability (%)
Ha et al. 2016 [19]	Korea	Fruit	Methanol extraction	Polyphenolic (mg GAE/g) Flavonoids (mg/L)	90 ± 3.5 51 ± 2.1	In vitro experiments on B16F10 and HDF cell lines	Cell viability (%) Matrix metalloproteinase (MMP-1) protein expression (relative protein level) Phospho-extracellular signal-regulated kinase (p-ERK) protein expression (relative protein level)
Halm et al. 2010 [15]	Korea	Fruit	Methanol extraction	—	—	In vitro experiments on U87MG cell line	Cell viability (%) Cell death on apoptosis (%)
Halm et al. 2011 [11]	Korea	Stem	OHs were blended and freeze-dried into powder	Dry matter (%) Ash (%) Crude protein (%) Crude fate (%) Crude fiber (%) Nitrogen-free extract (%)	98.34 ± 0.04 17.20 ± 0.06 10.75 ± 0.02 3.53 ± 0.07 10.04 ± 0.05 56.82 ± 0.05	Oral administration on Sprague–Dawley rats	Body weight (g) Glucose (mg/dL) Total cholesterol (mg/dL) Triglyceride (mg/dL) High-density lipoprotein cholesterol (HDL-C) (mg/dL) Free fatty acid (μEq/L)
Halm et al. 2015 [7]	Korea	Fruit and stem	Hexane and ethyl acetate extractions	Polyphenolic (mg GAE/g) Flavonoid (mg QE/g)	5.05 ± 0.28 1.91 ± 0.29	In vitro experiments on HeLa cell line Xenograft on mice	Cell viability (%) Tumor weight (g)
Halm et al. 2016 [23]	Korea	Fruit	Methanol extraction	—	—	In vitro experiments on AGS cell line	Cell viability (%) Cell death on apoptosis (%)
Han et al. 2018 [20]	Korea	Fruit	Water extraction	—	—	In vitro experiments on HDF cell line	Cell viability (%) Collagen synthesis (%) MMP-1 protein expression (relative protein level) p-ERK protein expression (relative protein level)
Hwang et al. 2011 [21]	Korea	Stem and peel	Ethanol and water extraction	—	—	In vitro experiments on MC3T3-E1 cell line	Cell viability (%) Collagen synthesis (%)
Jung et al. 2012 [25]	Korea	Stem	Methanol and hexane extraction	—	—	In vitro experiments on MCF-7 cell line	Cell viability (%)
Kang et al. 2013 [13]	Korea	Stem	OHs were blended and freeze-dried into powder	Moisture (% w/w) Ash (% w/w) Carbohydrate (g/100g) Crude protein (g/100 g) Crude fat (g/100 g) Fiber (g/100 g) Fe^2+^ (mg/g) Ca^2+^ (mg/100 g)	2.9 ± 1.2 13.8 ± 1.4 46.6 ± 5.2 4.9 ± 0.5 3.1 ± 0.7 28.9 ± 3.4 5.8 ± 1.3 2931.3 ± 10.2	Oral administration on mice	Body weight (g) Glucose (mg/dL) Insulin (ng/dL) Total cholesterol (mg/dL) Triglyceride (mg/dL) Free fat acid (μEq/L) HDL-C (mg/dL)
Kim et al. 2013 [22]	Korea	Stem	Methanol and acetone extraction	Methanol extraction Polyphenolic (mg GAE/g) Flavonoids (mg/L) Acetone extract Total phenolics (mg/g) Flavonoids (mg/L)	3.4 ± 0.3 91.1 ± 0.6 3.1 ± 0.2 124.6 ± 0.0	In vitro experiments on SW480 and MCF-7 cell lines	Cell viability (%)
Lee et al. 2013 [6]	Korea	Fruits	OHs were blended and freeze-dried into powder	—	—	In-vivo experiments on HR-1 mice	Tumor weight (g)
Moon et al. 2020 [5]	Korea	Stem	Microwave-assisted extraction	Polyphenolic (mg GAE/g) Flavonoids (mg/L)	33.1 ± 2.7 4.06 ± 0.6	In vitro experiments on HaCaT cell line	Cell viability (%) MMP-1 protein expression (relative protein level) p-ERK protein expression (relative protein level)
Yang et al. 2018 [9]	Korea	Stem	Water extraction	Arabinose (%) Galactose (%) Xylose (%) Neutral sugar (mg/g) Uronic acid (mg/g) Protein (mg/g)	40.3 ± 0.0 41.3 ± 0.1 13.6 ± 0.0 528.2 ± 3.5 351.6 ± 4.3 23.9 ± 1.4	Oral administration on mice fed with a high-fat diet	Body weight (g) Total cholesterol (mg/dL) Triglyceride (mg/dL) Insulin (ng/mL)
Yoon et al. 2009 [26]	Korea	Fruit	Water extraction	Polyphenolic (mg GAE/g) Flavonoids (mg/L)	4.49 ± 0.13 1.31 ± 0.0	In vitro experiments on MCF-7 cell line	Cell viability (%) Cell death on apoptosis (%)

**Table 2:** Risk of bias rating of individual studies.

Study	Random Sequence Generation	Allocation Concealment	Selective Reporting	Blinding of Participants	Blinding of Outcome Assessment	Incomplete Outcome Data
Cho et al. 2005 [24]						
Ha et al. 2016 [19]						
Halm et al. 2010 [15]						
Halm et al. 2011 [11]						
Halm et al. 2015 [7]						
Halm et al. 2016 [23]						
Han et al. 2018 [20]						
Hwang et al. 2011 [21]						
Jung et al. 2012 [25]						
Kang et al. 2013 [13]						
Kim et al. 2013 [22]						
Lee et al. 2013 [6]						
Moon et al. 2020 [5]						
Yang et al. 2018 [9]						
Yoon et al. 2009 [26]						
Risk of bias rating		Low risk of bias		High risk of bias		Uncertain

Green color: Low risk of bias; Blue color: High risk of bias; Yellow color: Uncertain

**Table 3:** Summary of standardized mean differences (SMDs) comparison between OH treatment and control groups.

Medical Applications	No. of Studies	Comparison Perspectives	n	SMD (95% CI)	I^2^	Total Effect (SMD and I^2^)
Controlling skin aging	4	Cell viability (%)	10	−0.97 [−1.97, 0.04]	0%	0.29 [−0.55, 1.13] I^2^ = 54%
		MMP expression	10	0.88 [−2.07, 3.84]	74%	
		p-ERK expression	10	0.51 [−0.97, 1.98]	41%	
		Collagen synthesis (%)	9	1.18 [0.11, 2.26]	0%	
Inhibition of cancer growth	9	Cell viability (%)	43	−2.05 [−2.73, −1.37]	6%	−1.17 [−1.72, −0.62] I^2^ = 69%
		Cell death on apoptosis (%)	14	2.17 [1.01, 3.34]	0%	
		Tumor weight (g)	8	−2.63 [−4.25, −1.02]	0%	
Inhibition of diabetes	3	Body weight (g)	34	3.33 [0.16, 6.50]	96%	−0.96 [−2.09, 0.16] I^2^ = 95%
		Total cholesterol (mg/dL)	34	–0.88 [−13.02, −0.44]	86%	
		Triglyceride (mg/dL)	34	–6.73 [−13.02, −0.44]	93%	
		Glucose (mg/dL)	28	–11.89 [−16.49, −7.28]	69%	
		Insulin (ng/mL)	34	−0.03 [−3.36, 3.30]	96%	
		Free fatty acids (FFA) (μEq/L)	28	−0.44 [−2.25, 1.38]	89%	
		HDL-C (mg/dL)	28	0.82 [0.04, 1.60]	39%	

## Supplementary Materials

The following supporting information can be downloaded at: https://www.mdpi.com/article/10.3390/antiox12010174/s1, Table S1: Characteristics of individual studies for subgroup analysis.
