# Supplementary material for: Beneficial Effects of Opuntia humifusa (Korean Cheonnyuncho) on Human Health Based on Antioxidant Properties: Systematic Review and Meta-Analysis
Source: Antioxidants (Basel). 2023 Jan 11;12(1):174. doi: 10.3390/antiox12010174 (PMC9854510; doi:10.3390/antiox12010174)
Supplement: Supplementary file 1 [file antioxidants-12-00174-s001.zip › antioxidants-2132188-supplementary.pdf]

Supporting Information

Beneficial Effects of *Opuntia hunifusa* (Korean Cheonnyuncho) on Human Health Based on Antioxidant Properties: Systematic Review and Meta-analysis

Le Thi Nhu Ngoc[1], Ju-Young Moon[2,*], and Young-Chul Lee[2,*]

[1]Department of Industrial and Environmental Engineering, Graduate School of Environment, Gachon University, 1342 Seongnam-Daero, Sujeong-Gu, Seongnam-Si, Gyeonggi-Do 13120, Republic of Korea

[2]Department of Beauty Design Management, Han-sung University, 116 Samseongyoro-16gil, Seoul 02876, Republic of Korea

[3]Department of BioNano Technology, Gachon University, 1342 Seongnam-Daero, Sujeong-Gu, Seongnam-Si, Gyeonggi-Do 13120, Republic of Korea

*Correspondence: dreamdbs@gachon.ac.kr; Tel.: +82-31-750-8751; +82-31-750-4748

Table S1. Characteristics of individual studies for subgroup analysis

Reference	Raw materials	Extraction methods	Cancer cell lines	Concentration of OH extract (μg/mL)	Treatment		Control		n
					Mean	SD	Mean	SD	
Halm *et al.* 2015	Fruit	Hexane	Hela cells	100	68	6.1	110	9.2	4
		Ethyl acetate	Hela cells	100	75	5.1	125	8.4	4
Kim *et al.* 2013	Stem	Ethyl acetate	SW480	100	80.2	3.9	100	5.5	3
			SW480	100	24.3	5.1	100	5.5	3
		Acetone	MCF-7	100	54.4	9.1	100	6.8	3
			MCF-7	100	21.1	1.2	100	3.8	3
	Fruit	Methanol	AGS	100	34.5	8.5	100	5.1	4
			U87MG	100	82	6.9	100	7.3	3
			U87MG	500	51	5.5	100	15.3	3
Cho *et al.* 2005	Stem	Methanol	RAW 264.7	100	82	6.9	100	7.3	4
	Stem	Hexane	RAW 264.7	100	97	6.1	100	5.8	4
	Stem	Ethyl acetate	RAW 264.7	100	98	4.5	100	3.6	4
	Stem	Water	RAW 264.7	100	98	3.8	100	4.9	4
Hwang *et al.* 2011	Stem	Ethanol	MC3T3-E1	100	110	4.7	118	5.3	3
		Water	MC3T3-E1	100	118	5.9	120	7.2	3
Jung *et al.* 2012	Stem	Methanol	MCF-7	100	88	10.2	100	6.4	4
		Hexane	MCF-7	100	87	3.9	100	5.9	4
Yoon *et al.* 2009	Fruit	Water	MCF-7	100	85	5.7	98	10.2	3
